# miR-29s: a family of epi-miRNAs with therapeutic implications in hematologic malignancies

**DOI:** 10.18632/oncotarget.3805

**Published:** 2015-04-12

**Authors:** Nicola Amodio, Marco Rossi, Lavinia Raimondi, Maria Rita Pitari, Cirino Botta, Pierosandro Tagliaferri, Pierfrancesco Tassone

**Affiliations:** ^1^ Department of Experimental and Clinical Medicine, Magna Graecia University and Medical Oncology Unit, T. Campanella Cancer Center, Salvatore Venuta University Campus, Catanzaro, Italy; ^2^ Laboratory of Tissue Engineering-Innovative Technology Platforms for Tissue Engineering (PON01-00829), Rizzoli Orthopedic Institute, Palermo, Italy; ^3^ Sbarro Institute for Cancer Research and Molecular Medicine, Center for Biotechnology, College of Science and Technology, Temple University, Philadelphia, PA, USA

**Keywords:** miR-29a, miR-29b, miR-29c, hematologic malignancies, multiple myeloma

## Abstract

A wealth of studies has highlighted the biological complexity of hematologic malignancies and the role of dysregulated signal transduction pathways. Along with the crucial role of genetic abnormalities, epigenetic aberrations are nowadays emerging as relevant players in cancer development, and significant research efforts are currently focusing on mechanisms by which histone post-translational modifications, DNA methylation and noncoding RNAs contribute to the pathobiology of cancer. As a consequence, these studies have provided the rationale for the development of epigenetic drugs, such as histone deacetylase inhibitors and demethylating compounds, some of which are currently in advanced phase of pre-clinical investigation or in clinical trials. In addition, a more recent body of evidence indicates that microRNAs (miRNAs) might target effectors of the epigenetic machinery, which are aberrantly expressed or active in cancers, thus reverting those epigenetic abnormalities driving tumor initiation and progression. This review will focus on the broad epigenetic activity triggered by members of the miR-29 family, which underlines the potential of miR-29s as candidate epi-therapeutics for the treatment of hematologic malignancies.

## INTRODUCTION

Genetic alterations, including point mutations, chromosomal translocations, amplifications and deletions, are hallmarks of cancer. These events may lead to oncogene activation, formation of chimeric oncoproteins and/or inactivation of tumor suppressor (TS) genes [1]. The contribution of such aberrations to the pathogenesis of different hematologic malignancies is now well established [2, 3]. Nevertheless, increasing knowledge in cancer epigenetics [4], as well as the availability of experimental tools to study epigenetic modifications [5], suggest that epigenetic alterations are also involved in the development of human cancer [6].

Epigenetics is defined as the study of heritable changes in gene expression and chromatin structure due to chemical modifications which occur independently of changes in DNA primary sequence [7]. A number of epigenetic modifications have been so far characterized, and among these, post-translational modification of histones [8], DNA methylation [9, 10] and the most recently discovered non-coding RNAs [11] are currently acknowledged as key determinants in the pathogenesis of human cancer, generally because aberrant epigenetic modifications turn on critical oncogenic pathways which drive tumor development and/or progression [12]. In contrast to genetic mutations, epigenetic changes are potentially reversible and this notion has prompted the design and further development of “epi-therapeutics”, such as demethylating drugs and histone deacetylase (HDAC) inhibitors, some of which are FDA-approved for the treatment of hematologic malignancies [13].

An emerging class of epigenetic regulators is represented by microRNAs (miRNAs), a class of small non-coding RNAs, which regulate gene expression at the post-transcriptional level and with critical roles in fine-tuning a wide array of biological processes [14]. miRNAs are small ncRNAs, only 20–22 nucleotides (nt) long. A single miRNA can control hundreds of different proteins produced in the cell by regulating their mRNA translation and stability via recognition of complementary target sites in their 3′UTR [15]. The biogenesis of miRNAs, their regulation and mode of action have been extensively addressed elsewhere and will not be discussed in detail in this review. MiRNAs regulate most of basic processes in cells such as proliferation, metabolism and apoptosis [16], and they are central in determining fate specification of a developing cell [17]. Noteworthy, miRNAs are dysregulated in almost all hematologic malignancies [18]; those up-regulated in cancer cells generally contribute to carcinogenesis by inhibiting tumor suppressor genes and are considered oncogenic miRNAs (OncomiRs) [19], while down-regulated miRNAs generally behave as TS-miRNAs and target oncogenes [14]. Silencing OncomiRs with miRNA inhibitors [20-23] or replacing TS-miRNAs with synthetic miRNA mimics [24-30] is demonstrating a valuable experimental strategy for the treatment of hematologic malignancies such as multiple myeloma (MM) [31-36]. MiRNAs endowed with tumor suppressive or anti-angiogenetic activity within the microenvironment or with synergistic activity with chemotherapeutic agents indeed represent promising tools to develop translational miRNA-based therapeutic approaches [25, 30, 37-40].

A subclass of miRNAs, named “epi-miRNAs”, which target epigenetic regulators such as DNA methyltransferases (DNMTs), HDACs or components of the polycomb repressor complexes, have proven to contribute to the epigenetic cellular landscape and represent novel tools to revert aberrant epigenetic alterations commonly found in cancer [41]. Among a variety of miRNAs, the miR-29 family represents the prototypical example of epi-miRNAs, since miR-29s have been demonstrated to target a number of epigenetic effectors thus inhibiting their aberrant expression and activity and leading to the re-activation of relevant oncosuppressive pathways in hematologic malignancies [28, 42, 43].

Here, we aim at reviewing the most common epigenetic abnormalities found in cancer, focusing on recent advancements on miR-29 family members as novel epigenetic regulators. Altogether, these studies underscore the potential of miR-29-based therapeutic approaches for the treatment of hematologic malignancies.

## EPI-MIRNAS

The expanding knowledge of the epigenome in cancer has highlighted the pivotal role that aberrant epigenetic regulation plays in the pathogenesis of many hematologic malignancies [44]. Most of the epigenetic mechanisms occur at the level of chromatin, the higher order structure of DNA. Chromatin is built up by nucleosomes which contain ±146 bp of DNA wrapped around an octamer of four core histones (H2A, H2B, H3 and H4). The chromatin structure and compactness relies upon epigenetic mechanisms including DNA methylation, post-translational modifications of histones and nucleosome positioning. The dynamics of such modifications affect the accessibility of the transcriptional machinery towards chromatin regions and ultimately leads to gene expression modification [45]. Epigenetic modulators basically regulate two processes: DNA methylation and histone modification. A brief overview of the key molecular mechanisms underlying these processes is provided in BOX-1 and BOX-2.

In the last few years, a wealth of studies has demonstrated that epi-miRNAs might control the epigenetic landscape of cancer by targeting key epigenetic effectors, such as DNMTs, HDACs or polycomb genes [41]. Manipulation of epi-miRNAs indeed produces profound consequences in the cellular epigenome, by affecting the expression of epigenetically-regulated genes involved in multiple cellular pathways.

The field of epi-miRNAs thus appears very promising and the literature relative to aspects in cancer detection, prognosis and therapeutic value of such miRNAs is continously rising.

A number of miRNAs, including miR-148a, miR-152 [46] and miR-222 [47] has been described to directly target the mRNA for DNMTs at 3′UTR. Downregulation of DNMT1 by miR-148a and miR-152 leads to re-expression of hypermethylated tumor suppressor genes, like Rassf1a and p16INK4a [48]. Importantly, some of these miRNAs, like miR-148a and miR-199a, are themselves regulated by methylation in cancer cells [49, 50], thus generating feedback regulatory loops which tightly connect miRNAs and DNA methylation pathway. In hepatocellular [51], endometrial cancer [52] and osteosarcoma [53], the tumor suppressive miR-101 targets EZH2, the catalytic subunit of the PRC2 (Polycomb Repressive Complex 2) complex, which is responsible of the H3K27me3-mediated silencing of tumor suppressor genes in cancer [54]. Moreover, other miRNAs, like miR-26a, miR-214 and miR-32 [55] have been described to target EZH2 gene. Importantly, a novel signaling network consisting of Specificity Proteins (SP), oncogenic miRNAs belonging to the miR-17-92, miR-106b-25 and miR-106a-363 clusters and ZBTB4 has been found to control EZH2 expression, and modulation of this gene/miRNA network represents a novel therapeutic approach for treatment of breast cancer [56] and possibly of other malignancies. In aggressive B-cell lymphomas, c-MYC-mediated recruitment of EZH2 causes H3K27me3-dependent silencing of the tumor suppressor miR-26a, and c-MYC or EZH2 inhibitors reactivate miR-26a expression in this malignancy [57]. MiR-15a/16-1 [58], miR-203 [59], miR-128 [60, 61], miR-194 [62] and miR-200c [63] instead control the PRC1 complex activity by targeting the Bmi-1 subunit. MMSET (MM SET domain), also known as WHSC1 (Wolf-Hirschhorn syndrome candidate 1) or NSD2 (nuclear SET domain-containing 2) is a SET-domain containing HMTase (histone methyltransferase) that is involved in the recurrent chromosomal translocation t(4;14) resulting in IgH enhancer-driven overexpression of MMSET in up to 20% of MM [64]. MMSET binds to transcriptionally active regions of the genome and specifically catalyzes H3K36 dimethylation, a mark associated with regions of open chromatin [65]. The oncogenic functions of MMSET in MM are associated with its HMTase catalytic activity making it amenable to therapeutic intervention. Recent reports suggest that like EZH2, MMSET is overexpressed in diverse solid tumors, however the consequence and the mechanism of this overexpression are not completely understood [65, 66].

Notably, EZH2 and MMSET have been found coordinately overexpressed and associated with human progression in some tumors. Importantly, EZH2 was found to repress the transcription of several miRNAs by H3K27me3 [67]; among 15 selected EZH2-repressed miRNAs, miR-26a, miR-31 and miR-203, that bind to multiple sites in the 3′UTR of MMSET mRNA, were found to cause a decrease in MMSET protein expression. These results indicate that EZH2 is able to activate MMSET activity by a miRNA network and that EZH2-MMSET HMTase axis coordinately functions as a master regulator of transcriptional repression, activation, and oncogenesis [68]. On the other hand, MMSET is able to enhance the proliferation of MM cells by stimulating the expression of c-MYC at the post-transcriptional level: this occurs by increased trimethylation of H3K9 and decreased H3 acetylation of miR-126*, which results in miR-126* down-regulation and consequent increase in its target c-MYC [69]. Of note, the biological scenario involving MMSET in tumor cell proliferation appears even more complicated on the light of the discovery of the novel box H/ACA ncRNA, ACA11, encoded within intron 18–19 of the *WHSC1* gene. ACA11 is an orphan small nucleolar RNA (snoRNA) that binds to a novel small nuclear ribonucleoprotein (snRNP) complex in MM cells. Overexpression of ACA11 protected MM cells from oxidative stress and modulated tumor proliferation, and knockdown of ACA11 slowed cell proliferation and sensitized MM cells to cytotoxic chemotherapy. These results further contribute to shed light on the prominent role played by non-coding RNAs in MMSET-driven epigenetic modifications [70].

The type II arginine methyltransferases PRMT5, that catalyze ω-N^G^-monomethylation and ω-N^G^,N^G^′-symmetric dimethylation [71], is overexpressed in lymphoid cancer cell lines and its level correlates with increased symmetric methylation of histones H3R8 and H4R3. Pal and colleagues demonstrated that PRMT5 overexpression in mantle cell lymphoma was dependent on down-regulation of the PRMT5-targeting miRNAs miR-92b and miR-96 [72]; in leukemia and lymphoma cells, PRMT5 was found to be also targeted by miR-19a, miR-25, miR-32 and miR-197 and to inhibit the expression of TS belonging to Rb family [73].

Enzymes regulating histone acetylation may also be targeted by epi-miRNAs. Some authors demonstrated that miR-9* is down-regulated in Waldeström Macroglobulinemia (WM) cells where it targets HDAC4, and miR-9* reconstitution increases the levels of acetylated histone H3 and dampens tumor growth [74]. miR-34a, a tumor suppressor in solid and hematologic cancers [24, 29, 36, 75-78], elicits HDAC-inhibitor activity by targeting HDAC1 and HDAC7 [79]. Interestingly, members of miR-200 family, which are able to revert aberrant histone acetylation in hepatocellular and lung carcinomas by targeting HDAC4, are in turn down-regulated by HDAC4 in an SP1-dependent manner [80]. These findings again support the existence of regulatory loops which epigenetically modulate gene expression. A comprehensive list of the most studied epi-miRNAs along with their epigenetic targets is reported in Table 1.

**Table 1:** The most representative cancer-related epi-miRNAs and their targets

epi-miRNA	Epigenetic target(s)	Cancer	Reference
miR-29a/b/c	DNMT3A, DNMT3B	AML, MM, NSCLC	[28, 42, 81]
miR-148a/b	DNMT1	GC, HC	[46, 184, 185]
miR-152	DNMT1	GC, BC, OC, NB	[46, 186, 187]
miR-217	DNMT3A	CML	[188]
miR-1741 miR-16c miR-222 miR-1632	DNMT3A DNMT3B	OC	[47]
miR-185	DNMT1	TN-BC, OC	[186, 189]
miR-140	DNMT1	HC	[190]
miR-342	DNMT1	CC	[191]
miR-143	DNMT3A	CML	[192]
miR-199a-3p	DNMT3A	TC	[193]
miR-9*	HDAC4 HDAC5	WM	[74]
miR-34a	HDAC1 HDAC7	BC	[79]
miR-874	HDAC1	HN	[194]
miR-520h	HDAC1	GC	[195]
miR-449a	HDAC1	PC	[196]
miR-145	HDAC2	HC	[197]
miR-206	HDAC4	GC	[198]
miR-200a	HDAC4	HC	[199]
miR-200b	SUZ12	BC	[200]
miR-101	EZH2	Endometrial cancer	[52]
miR-101	EZH2	GC	[201]
miR-26a, miR-214, miR-32	EZH2	Oral cancer GC	[55] [202] [203]
miR-15a/miR-16	Bmi-1	OC	[58]
miR-203	Bmi-1	EC	[59]
miR-200c	Bmi-1	Melanoma	[63]
miR-128	Bmi-1	BC	[61]
miR-26a, miR-31, miR-203	MMSET	PC	[68]

Abbreviations: AML, Acute Myeloid Leukemia; BC, Breast Cancer; CML, Chronic Myeloid Leukemia; EC, Esophageal Cancer; GC, Gastric Cancer; HC, Hepatocellular Carcinoma; HN, Head and Neck Cancer; MM, Multiple Myeloma; NB, Neuroblastoma; NSCLC, Non Small Cell Lung Cancer; OC, Ovarian Cancer; PC, Prostate Cancer; TN-BC, Triple Negative Breast Cancer.

The first evidence supporting the existence of epi-miRNAs was achieved in lung cancer experimental models, where miR-29b was proven to directly targets DNMT3A and DNMT3B [81]. MiR-29b is nowadays widely acknowledged as TS in a variety of solid tumors, like cholangiocarcinoma [82], osteosarcoma [83], hepatocellular [84] and colorectal carcinoma [85], uterine leiomyoma [86], ovarian carcinoma [87] and many others.

Notably, a still raising number of studies indicates that members of the miR-29 family behave as epi-miRNAs by down-modulating important drivers of the epigenetic machinery. Recently, by exploiting molecular profiles of more than 3,000 tumors from 11 human cancer types in “The Cancer Genome Atlas”, Jacobsen et al. systematically analyzed expression of miRNAs and mRNAs to infer recurrent cancer-associated miRNA-target relationships: importantly, the miR-29 family was found to regulate the DNA methylation pathways [88], thus strengthening the importance of miR-29s as key epigenetic regulators in cancer.

On these premises, we here review the most recent findings supporting the emerging role of miR-29s as key negative regulators of tumor cell growth and survival as well as their relevance as prognostic factors in the hematologic neoplasias where they have been studied, providing an overview of their genomic organization, of the molecular mechanisms controlling their expression and of their ability to affect the epigenome of cancer cells, which ultimately lend support to their role as TS epi-miRNAs.

## MIR-29 FAMILY

The human miR-29 family consists of 3 members: miR-29a, miR-29b and miR-29c. The structure, function, and regulation of miR-29s are in high degree homologues in human, mouse and rat. MiR-29a was initially detected in HeLa cells by Lagos-Quintana [89], followed by the subsequent discovery of miR-29b and miR-29c [90, 91]. The precursors of miR-29 family are transcribed in 2 bi-cistronic clusters: miR-29a/b-1 cluster is located on chromosome 7 (7q32), while miR-29b-2/c cluster is located on chromosome 1 (1q32). Even if miR-29b-1 and miR-29b-2 are located in different parts of genome, they both have identical mature sequences; indeed, mature miR-29a and miR-29c are identical except for only a single nucleotide outside the seed sequence. However, despite the identical seed sequence, recent studies suggest a context-dependent pattern for miR-29s expression and regulation. Consistently, a variety of physiological and disease conditions have been correlated to a differential expression and subcellular organization of miR-29 family members. In 2007, it has been shown that miRNAs can also localize inside the nucleus; in this study, authors also demonstrated a different subcellular distribution of miR-29s, supporting the hypothesis that their functional relevance may not be identical. While miR-29a mainly localizes in cytoplasm, miR-29b and miR-29c are found enriched in the nucleus, this nuclear localization being correlated to the presence of the AGUGUU motif at the 3′ end of miR-29b, which allows the transport inside the nucleus where it can act as transcriptional or splicing factor [92].

### Regulation of miR-29s expression

Several reports have shown that the expression of miR-29s is dysregulated in cancer cells and the mechanisms responsible for this phenomenon is still a matter of investigation. Altogether, these studies indicate that members of miR-29 family are regulated in both a transcriptional and post-transcriptional fashion.

### Transcriptional regulation

Different binding sites for several transcriptional factors have been identified in the promoter of miR-29a/b-1 and miR-29b-2/c clusters, thus explaining the inconsistent ratio of miR-29a, miR-29b and miR-29c expression across the tissues [93]. The promoter region of both miR-29 clusters contains a putative E-box Myc binding site and increased expression of c-MYC, such as in B-cell lymphoma, contributes to suppress miR-29s expression [94]. Several studies indicate that oncogenic transcription factors act by repressing miR-29b expression. MiR-29b-1/a promoter also contains a putative binding site for Gli, a hedgehog signalling component, as well as multiple NF-kB sites [95, 96]. Four Ying-Yang-1 (YY1) binding sites associated with transcriptional repressor factors Polycomb group (PcG) Ezh2 and HDAC1 are localized in miR-29b-2/c on chromosome 1; NF-kB negatively regulates miR-29 expression by acting on the YY1/PcG complex which in turn causes epigenetic repression of miR-29b-2/c transcription in progenitor muscle cells. Epigenetic mechanisms have been found to affect miR-29b levels in hematologic malignancies, like acute myeloid leukaemia (AML) and MM. Evidence supporting such regulation will be discussed in the section describing the role of miR-29b in the context of each hematologic malignancy.

**Figure 1:**
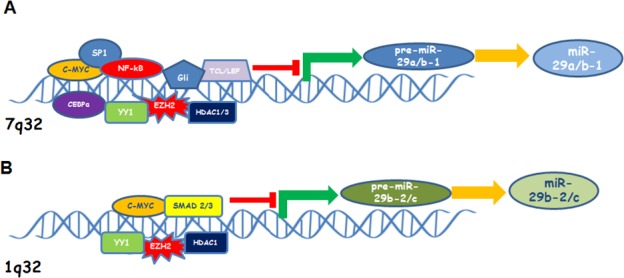
Cartoon showing the most relevant transcription factors and co-factors that inhibit miR-29 a/b-1 (**A**) or miR-29b-2/c (**B**) expression.

MiR-29b-1/a promoter region also includes CCAAT/enhancer-binding protein-α (CEBPA) binding site. The transcription factor CEBPalpha regulates miR-29 expression directly, and CEBPalpha loss in AML leads to silencing of miR-29b [97]. Canonical Wnt signaling induces miR-29a expression by two T-cell factor/lymphoid enhancer factor (TCF/LEF) binding sites in the miR-29b-1/a promoter; this effect is necessary for osteoblast differentiation targeting antiosteogenic factors and extracellular matrix as well as Wnt signalling antagonist [98]. Mir-29b-1/a promoter region contains 5 GAS (IFN-γ-activated sequences) elements; these binding sites are recognized by the interferon-γ induced STAT1, ultimately leading to increase of miR-29a and miR-29b expression. In melanoma, increased expression of miR-29s upon interferon-γ activation of STAT1, results in diminished expression of CDK6 and cell cycle arrest in the G1 phase [99]. Transforming growth factor-β (TFG-β)-Smad-induced miR-29 deregulation is characteristic of fibrosis process and carcinogenesis [100]. TFG-β activates SMAD2/3 which in turn binds Smad Binding Element (SBE) contained inside the promoter region of miR-29b-2/c; in addition, TFG-β also inhibits Smad7, an inhibitor of Smad2/3. Interestingly, Smad3 binding to SBE elements interferes with the MyoD binding to the E-box sites that lies in close proximity and permits YY1 binding to Ezh2 and HDAC1 complex thus inducing a strong miR-29 repression [101]. A cartoon illustrating the most relevant transcription factors or co-factors that regulate miR-a/b-1 or miR-29b-2/c is reported in Figure 1.

### Post-transcriptional regulation

While miRNA expression can be regulated at any step during their biogenesis, very little is known regarding the decay of miRNAs.

Some studies showed the heterogeneity of miRNA lifespan, ranging from many days *in vivo* to few hours in cell culture [102]. MiR-29s are also regulated in a post-transcriptional manner. An interesting study [92] in HeLa cells showed that even if the clusters of miR-29b-1/a and miR-29b-2/c were co-transcribed, the mature miR-29s show differential expression. In detail, while miR-29a was expressed in all stages of cell cycle, miR-29b was rapidly degraded in cycling cells but was stable in mitotic cells and finally miR-29c was even not detected. Studies on the turnover rate of miR-29s demonstrated that uracils at nucleotide positions 9-11 of miR-29b are important for its rapid turnover, while difference between miR-29a and miR-29c result in the relative quicker decay of miR-29c [92, 102].

## MIR-29S IN NORMAL HEMATOPOIESIS AND IMMUNE RESPONSE

The differentiation of normal hematopoietic cells is a highly regulated process in which pluripotent hematopoietic stem cells (HSCs) give rise to all the different cells that form the blood and the immune system. This complex process is driven by an intricate network of transcriptional and post-trascriptional mechanisms which need to be carefully regulated. In the recent past, different studies evidenced the role of miRNAs as master regulators of blood cells differentiation and function by influencing lineage commitment, proliferation, activation and death [103]. The development of myeloid and lymphoid cells depends on the activation of specific genetic programs that are responsible for the reduction in cell proliferation and the expression of lineage specific genes. miRNAs provide an additional level of control beyond transcription factors by fine-tuning differentiation and adjusting the cell response to external stimuli. In particular, they play a crucial role in the regulation of blood cell specification by controlling the precise timing and expression levels of key factors and conferring robustness to regulative networks. To date, it has been shown that miRNAs are critical in almost every stage of hematopoiesis [104, 105]. miR-29a seems to have an important role in the early steps of hematopoiesis. Biessel et al. [106], using a combined characterization of miRNA and mRNA profiles, identified a different signature between CD133^+^ and CD34^+^CD133^−^ cells which can affect proliferation, prevent apoptosis, inhibit differentiation and cytoskeletal remodeling. In particular, an inverse correlation between miR-29a and both TPM1 and FZD5 in CD133^+^ cells was observed. TPM1 is an important component of the cytoskeleton with a key role in many aspects of eukaryotic cell behavior such as cell morphology, divisions, and motility while FZD5 is one of the seven-pass transmembrane Frizzled receptors interacting with Wnt proteins which regulate cell proliferation and stem cell maintenance. Additionally, miR-29 family plays an important role in physiological T cell differentiation by protecting the thymus against inappropriate involution caused by infections, through down-regulation of IFNAR1 [107]. Indeed, the thymic epitelium of miR-29a-deficient mice presented high levels of IFNAR1 that, in turn, increased sensitivity to pathogen associated molecular patterns (PAMPs). By targeting IFNAR1, miR-29a reduces the threshold at which thymic cells begin their involution processes, allowing this event to be triggered only in presence of an adequate amount of IFN-a/PAMPs. Beyond their role in immune cells development and maturation, miR-29 family has been reported to be involved in the modulation of immune response to inflammation and infection at different steps. A low level of miR-29 in NK cells, CD4+ and CD8+ T lymphocytes has been reported to be associated with an increased production and secretion of IFN-γ *in vitro* and, on the same line, mice with transgenic expression of a miR-29 sponge showed enhanced resistance to infection with *Listeria monocytogenes* or *mycobacterium bovis* and an overall reduced lung bacterial burden [108]. This effect appears to be dependent on the ability of miR-29 to negatively regulate both the T-box transcription factors T-bet and eomesodermin, and IFN-gamma production and secretion by T-helper cells [108, 109]. On the other hand, IFN-γ was found to upregulate miR-29 expression in T cells in a STAT1 dependent manner [99, 110]. Similarly, even type I interferons have been reported to upregulate miR-29 levels [99]. On this basis, it appears that miR-29s could tip the scales between pro and anti-inflammatory response being involved in a homeostatic regulatory loop in which IFN-γ induces miR-29 via STAT1 and miR-29, in turn, represses IFN-γ production. Due to its effect on IFN-γ biology, Smith et al [110] recently investigated the dynamic of miR-29 expression in different T helper subsets (Th1, Th2, Th17, Tregs) finding a general up-regulation of these miRNAs during differentiation, regardless of subtype. However, lymphocytes retrieved from miR-29a/b1-deficient mice exhibited an activated inflammatory phenotype *ex vivo*, producing significant higher levels of IFN-γ and IL-17 compared to wild-type mice. Along the same lines, miR-29a was recently found to inhibit the production by dendritic cells of IL-23, the most potent Th17 polarizing cytokine, by targeting directly IL-12p40 and indirectly IL-23p19, via reduction of ATF2, thus attenuating the pro-inflammatory Th17 T cell response *in vitro* [111]. Notably, we recently reported the capability of miR-29b to induce SOCS-1, one of the most important regulator of cytokines signaling, and to impair STAT3 and NF-kB signaling [26], thus suggesting that miR-29 is potentially endowed with anti-inflammatory properties, a feature which deserves further investigations.

Besides the canonical function of RNA interference, miR-29a and miR-29b have been reported to trigger inflammatory response and innate immune cells through binding to toll like receptors (TLR)-7/8 and induction of TNF-alpha and IL-6 production and secretion [112]. On this basis, miRNAs spontaneously released from different cells, including cancer cells, may act as paracrine modulators of both microenvironment and immune cells.

## MIR-29S IN HEMATOLOGIC MALIGNANCIES

Several reports indicate that the expression of miR-29 family members is widely deregulated in hematologic cancers and its reconstitution deeply impacts on the phenotype of cancer cells. Importantly, anti-tumor activity of miR-29s often relies on alterations of the cancer cell epigenome induced by their intrinsic epi-miRNA activity.

### Multiple myeloma

Multiple myeloma (MM) is a hematologic malignancy characterized by abnormal proliferation of plasma cells within the bone marrow. In the last years, the huge advancement in the understanding of its biology, the availability of investigational platforms for plasma cell discrasias [113-118] and novel agents [34, 115, 119-128] have significantly expanded the therapeutic perspectives. Moreover, emerging findings are disclosing the role of miRNAs in its pathogenesis and some of them likely represent valuable targets for therapeutics. In this context, the anti-MM activity of miR-29b has been extensively studied by our group. We first reported that miR-29b was significantly down-regulated in primary malignant plasma cells and MM cell lines. Enforced expression of miR-29b, achieved by either synthetic mimics transfection or by lentiviral-mediated delivery, triggered *in vitro* anti-MM activity since it inhibited cell growth and promoted apoptosis [27]. MCL1 and CDK6 suppression were demonstrated to be dependent by miR-29b targeting and likely responsible of cell growth inhibition and apoptotic triggering. Most importantly, *in vitro* data were recapitulated *in vivo* in a murine model of human MM consisting of MM xenografts stably expressing miR-29b. Importantly, harvested tumors revealed suppression of MCL-1 and CDK6 targets together with activation of caspase 3, thus linking the anti-tumor effects *in vivo* with miR-29b-dependent targeting. Synthetic miR-29b oligonucleotides delivered by different routes were also able to inhibit the growth of human MM xenografts in many preclinical models of MM, including the SCID-*synth-hu* system [114] which recapitulate the growth of MM cells in the context of the human BMM represented by BMSCs growing on a poli-ε-caprolactone scaffold which mimics the bone architecture [28]. To explore additional mechanisms of miR-29b action, we focused on the transcription factor SP1. In fact, high SP1 activity was previously shown to be relevant for sustaining survival and proliferation of MM [129] and WM cells [130]. Indeed, we demonstrated direct targeting of SP1 at 3′UTR along with the existence of a feed-back loop between SP1 and miR-29b, since both pharmacological or genetic inhibition of SP1 led to miR-29b transcriptional activation. Similar effects could be achieved after treating MM cells with the proteasome inhibitor bortezomib, which was previously shown to interfere with SP1/NF-kB pathway in AML cells [43]. Notably, bortezomib was able to affect the SP1/miR-29b loop by down-modulating SP1 expression and up-regulating miR-29b. These findings prompted to investigate the anti-MM activity of miR-29b mimics/bortezomib combination: indeed, enforced miR-29b in bortezomib-treated MM cells strongly increased growth inhibition and apoptosis in MM cells, while miR-29b inhibition by antagomiRs partially dampened bortezomib anti-MM activity. The relevance of miR-29b/SP1 loop was further confirmed by the observation that the PI3/AKT pathway, which is hyperactivated in MM, can partially rescue miR-29b-mediated anti-MM activity *in vitro*. These data highlighted the potential utility of miR-29b as an innovative therapy for MM and likely as an useful tool to overcome emerging resistance to bortezomib in relapsed /refractory MM patients. As a further support to these findings, a recent report identified PSME4 as a novel target of miR-29b/c in MM cells [131]. PSME4 encodes for the proteasome activator PA200. In the presence of miR-29b, PA200 was reduced reinforcing the proteasome inhibition induced by bortezomib. miR-29b was also capable to increase protein aggregates within the cytoplasm of MM cells, without leading to aggresome/autophagosome formation, which are critical for adequate misfolded protein elimination. The overall effect was a potentiation of the apoptotic effects of bortezomib.

Circulating serum miRNAs might serve as cancer biomarkers [32]. Sevcikova and colleagues demonstrated that miR-29a was present in the blood serum and expression of miR-29a can differentiate between healthy subjects and MM patients [132]. However, results are very preliminary and further studies are required to assess the potential of circulating miR-29s as biomarkers in MM and other malignancies.

Several studies have shown that MM pathogenesis is strictly linked to epigenetic modifications [133], such as aberrant DNA hypermethylation. Interestingly, DNMT3A and 3B expression was higher in MM and plasma cell leukaemia (PCLs) patients and could be further raised in the presence of bone marrow stromal cells (BMSCs). By these data, we first demonstrated that miR-29b directly targets DNMT3A and 3B. A global methylation assay was then carried on MM cells to assess whether miR-29b could work as an epigenetic modifier. The results showed that miR-29b levels correlated with a robust reduction of global CpGs methylation in MM cells. To support the therapeutic relevance of our data, we demonstrated that miR-29b mimics potentiated the *in vitro* anti-MM activity of a widely acknowledged demethylating agent such as 5-azacytidine and confirmed DNMTs inhibition *in vivo*. Conversely, inhibition of DNMTs in MM cells did not cause any change in miR-29b levels, thus indicating that methylation of miR-29b promoter does not account for the low miR-29 expression observed in MM [28].

Among the main mechanisms by which DNA methylation supports MM growth, inhibition of tumor suppressor genes (TSGs) due to promoter hypermethylation is highly relevant [134]. As miR-29b interferes with DNA methylation, we sought to explore whether epigenetic suppression of TSGs could be hampered by this miRNA. Among TSGs, SOCS-1 has been documented as down-regulated in human malignancies [135]. We checked the expression of SOCS-1 in MM and PCLs and found it consistently down-regulated as compared to normal plasmacells. A positive correlation between miR-29b and SOCS-1 was also unveiled in a specific clinical subgroup (TC2) of MM patients. Based on these findings, we demonstrated that miR-29b was a positive regulator of SOCS-1 by inducing demethylation of its promoter, the same effects being achieved by bortezomib-induced miR-29b induction. Either miR-29b mimics or bortezomib treatment hampered JAK/STAT signaling, the main pathway targeted by SOCS-1, and impaired migration of MM and endothelial cells [26]. Overall, these data unveiled more complex mechanisms of tumor suppressor activity exerted by miR-29b beyond direct targeting prediction and validation. Indeed, as an epi-miRNA, miR-29b widely enlarges the number of the targeted pathways within MM cells, thus explaining the consistent down-regulation that is observed across studies in both MM and primary PCLs.

### MM-bone disease

One of the hallmark of MM is an extensive bone damage due to the generation of multiple lytic lesions of the skeleton triggered by enhanced osteoclast (OCL) activity in combination with suppressed osteoblastic (OBL) function. This imbalance is induced by MM plasmacells interaction with BM microenvironment [136].

The current therapeutic approach of MM bone disease (BD) is based on the use of bisphosphonates (BPs) that promote OCL apoptosis [115, 123, 137-142]. However, BPs are associated with the development of serious adverse events in the mid- long term-treatments. Emerging evidence has highlighted a number of miR-mediated functions in BM microenvironmental cells, specifically osteoclasts (OCLs) and osteoblasts (OBLs), where deregulated miRNAs may impact on their differentiation, maturation and final activation. OCL formation requires permissive concentrations of M-CSF and RANK-L and is driven by contact with OBLs and BMSCs in the bone marrow. OCL precursors express RANK and the interaction between RANK-L and RANK is the major determinant of OCL formation [143, 144]. Epigenetic events are involved in the regulation of RANK-L expression in MM cells: Yan et al. recently demonstrated that TNFα in the BMM promotes RANK-L promoter demethylation, and this relies on a molecular circuitry which involves miR-140 and miR-126-3p, both known to repress DNMT1 translation [145].

Besides the increased OCL activity, the pathogenesis of MM BD is also related to a reduced OBL function. Wnt signalling and the transcription factor Runx2/Cbfa1 (Runt-related transcription factor 2/core-binding factor Runt domain α subunit 1) are both required for OBL differentiation. MiR-29b indeed plays a central role in bone remodelling [146] and Rossi et al. first identified its key role in the inhibition of OCL generation and function. In fact, miR-29b is progressively down-regulated during OCL differentiation of monocyte precursors cultured in the presence of M-CSF and RANK-L and restoration of miR-29b expression strongly suppresses the resorbing activity of OCL by reducing intracellular levels of tartrate acid phosphatase (TRAP), cathepsyn K, metalloproteinase type 2 and 9 (MMP-2 and MMP-9) [30]. The reduced expression levels of these specific OCL-resorbing enzymes is due to direct targeting of miR-29b on MMP-2 and c-FOS mRNAs. C-FOS is a key transcription factor for OCLs as it regulates the expression of the master OCL transcription factor, NFATC-1, which in turn encodes for critical OCL genes such cathepsin K, MMP9 and TRAcP. Another effect likely ascribed to c-FOS inhibition was an impaired rearrangement of actin rings, whose normal morphology is critical for OCL resorbing activity and bone adherence. Finally, we demonstrated that enforced expression of miR-29b reduces RANK expression on cell surface thereby limiting OCL capability to respond to RANK-L stimulation [30].

On the other hand, other authors had previously shown that miR-29b is up-regulated along osteoblastogenesis during late mineralization phases [146], showing its role as promoter of OBL differentiation and suggesting a potential mechanism of inhibition of collagen deposition. Evidence indicates that miR-29b also turns off the synthesis of key OBL-inhibitor proteins such as TGFβ3 and HDAC4, thus allowing the expression of Runx2, and down-regulates the Wnt pathway inhibitor CTNNBIP1 (catenin beta interacting protein 1) [147], thus confirming the epi-miR-29b activity also in this pathophysiological context. Although further studies are required to ascertain how miR-29b can impact on bone remodelling and how can be modulated to restore the physiologic balance between OBL/OCL within MM *milieu*, the findings above reported provide the preclinical evidence of miR-29b as novel potential therapeutic tool that may open a new scenario for the management of MM-BD and strongly encourage the study of miR-29b also in the context of other bone malignancies.

### Chronic Lymphocytic Leukemia

Chronic Lymphocytic Leukemia (CLL) has been, among malignant diseases, the first where miRNA deregulation was extensively studied. Calin et al. [148] compared the miRNome of 38 CLL samples with CD5^+^B cells from one normal lymph node and 5 healthy donors samples (2 tonsillar CD5^+^ B cells and 3 PBMCs) and highlighted a distinct signature for CLL. When considering distinct CLL subsets (IgVH mutated and unmutated), 5 miRNAs including miR-29c were identified as preferentially expressed in IgVH mutated CLLs. Therefore, miR-29c was associated with better prognosis in CLL. The same group asked whether the differentially expressed miRNAs at various stages may have an impact on CLL pathogenesis. To address this issue, 80 CLL samples [149] were separated into 3 distinct groups according to disease course and karyotype: indolent CLLs, aggressive CLLs with chromosome 11 deletion or without chromosome 11 deletion. These categories were chosen as the putative CLL oncogene TCL-1 was differentially expressed in the three CLL subgroups. Indeed, TCL1 deregulation has been considered as an early event for the switch from indolent to aggressive CLLs [150]. Putative TCL-1 targeting miRNAs were preliminary predicted by software analysis. Among these miRNAs, only miR-29b and miR-181b were found consistently down-regulated in aggressive CLL samples carrying ch.11 deletion. Target confirmation experiments demonstrated that TCL1 expression was reduced in the presence of miR-29b or miR-181b. Interestingly, as different miR-29 family members (miR-29a-2, miR-29b-2, miR-29c) were down-regulated in aggressive CLL forms, the authors concluded that this family has likely a central role in controlling the TCL-1 oncogene. These exciting data led investigators to develop a miR-29 transgenic mouse model [151]. In this model, a preferential expansion of CLL-like B cells (CD5+CD19+ B cells) was observed as compared to control. Interestingly, the course of the disease in the mice was indolent with only 20% of mice dying of leukaemia at old age. The peculiar finding that miR-29 family members are upregulated mostly in indolent CLLs, while their expression is reduced in aggressive CLL disease [152] provided an innovative tool for prognosis stratification of CLL patients and suggested that miR-29 may work as an oncogene and a tumor suppressor according to the disease stage [153], underpinning the multifaceted activity of miRNAs in tumor development and progression. miR-29 family members down-modulation is also likely to affect the regulation of apoptotic program in CLL cells. Indeed, MCL-1 is a validated miR-29b target in AML. MCL-1 has a central role in CLL progression and resistance to chemotherapy [154, 155]. Following these observations, emerging evidence indicated that miRNA changes within CLL cells may be also linked to BCR signalling and T cell-dependent stimuli [156]. CLL miRNA profile seems closely related to activated B cell mirnome. Indeed, BCR/co-stimulatory molecule ligation of B cells promotes miRNA changes in the same direction and magnitude of CLL cells. When considering miR-29 family, miR-29a/b/c can be up or down-regulated according to the specific BCR stimuli (anti IgM/CD40L or TLRs). Interestingly, B cell activation through anti IgM (BCR ligand) or CD40L (co-stimulatory molecule ligand) down-regulated miR-29c as observed in poor prognosis CLLs. The same group thus confirmed previous reports, where low levels of miR-29c were associated with unmutated, ZAP70+ CLLs, which carry a worse prognosis [152]. According to these data, the authors hypothesised that the disparities observed among previously published CLL miRNA signatures may be also due to differences in the activation status of CLL cells analysed other than different microarray platforms utilised.

An additional mechanism that modulates miRNA levels within CLL cells is represented by epigenetic silencing. Sampath et al. [157] asked whether HDAC activity could affect miRNA expression in CLL cells. Exposure to CLL cells to different HDAC inhibitors (LBH589, SAHA and MS275) partially restored the expression of miR-15, -16 and 29b, thus demonstrating an epigenetic control of miRNA expression. These findings indeed contribute to shed a new light on the mechanisms of action of HDAC inhibitors in cancer therapy.

Taking into account these data, CLL represents a paradigmatic disease to understand the function of miRNAs in cancer development and progression. Specifically, miR-29 family members seem to play a central role in determining the biological behaviour of malignant CLL B-cells.

### Acute myeloid leukemia

Acute myeloid leukemia (AML) is a heterogeneous disorder that includes many entities with diverse genetic abnormalities and clinical features. Abnormal proliferation, repression of apoptosis and differentiation blockade of hematopoietic stem/progenitor cells are widely accepted to be the main reasons leading to acute myeloid leukemia (AML). In addition to specific chromosomal translocations, deletions and amplifications (eg, t(15;17), −5q, −7q, +8), gene mutations (eg, *FLT3-ITD*, *CEBPA*, and *NPM1*) and oncogene deregulation are considered paradigm of this malignancy [158]. Results on miR-29 expression levels in such disease are quite controversial, this discrepancy depending on the different platforms used for the studies and on the different AML subtypes analyzed. Consistently, a diverse pattern of expression of miR-29 family members along with different biological functions is continuously emerging from the literature. miR-29a seems a *bona fide* oncogene in AML since enforcing its expression in immature mouse hematopoietic stem/progenitor cells results in the acquisition of self-renewal capacity by myeloid progenitors, biased myeloid differentiation, and the development of a myeloproliferative disorder progressing to AML [149]; the same authors demonstrated that homozygous deletion of the miR-29a/b-1 bicistron results in decreased numbers of hematopoietic stem and progenitor cells, decreased HSC self-renewal, and increased HSC cell cycling and apoptosis, such phenotype being dependent on the loss of miR-29a [159]. MiR-29a promotes progenitor cells proliferation by expediting G1 to S/G2 cell cycle transition; however, over-expression of miR-29b or miR-29c does not recapitulate the phenotype of miR-29a over-expression [160]. Conversely, Wang et al. found that miR-29a and miR-142-3p expression increases during myeloid differentiation of THP-1, NB4, and HL-60 AML cell lines and their over-expression promote myeloid differentiation both in cell lines and primary blasts. By *in silico* analysis, the authors found two genes, specifically CCNT2, a component of the positive transcription elongation factor b (P-TEFb), and CDK6 as putative targets of miR-29a, and functionally validated their down-regulation as important for miR-29a to induce myeloid and granulocytic differentiation [161]. Garzon et al. reported that a unique miRNA profile associated with the main cytogenetic and molecular subgroups of AML. Importantly, expression of *miR-29* family members was down-regulated in primary AML samples with the t(6;11) and t(9;11) translocations [162]. Moreover, primary CN-AML samples with wild-type nucleophosmin gene (*NPM1*) beared lower levels of miR-29s than CN-AML samples bearing *NPM1* mutations, thus indicating that miR-29 family may play a role in the pathogenesis of AMLs [163]. Lower levels of miR-29a were also found in MLL-rearranged pediatric AMLs as compared to AMLs carrying *NPM1* mutations or FLT3-ITDs, suggesting a role of this miRNA in the pathogenesis of a specific subgroup of leukemias. Following this pivotal study, others have reported significant lower expression of miR-29a/29b and higher expression level of two potential target genes, BCL-2 and MCL-1, in PBMCs from AML patients compared with control group. In addition, miR-29a expression in AML was significantly lower than that in CML [164].

Transcriptome analysis after ectopic transfection of synthetic miR-29b into leukemia cells indicates that miR-29b targets apoptosis, cell cycle and proliferation pathways. A significant enrichment for apoptosis genes, including MCL-1, was found among the mRNAs inversely correlated with miR-29b expression in 45 primary AML samples. AKT2 and CCND2 mRNAs were validated as targets of the miR-29 members, and the role of miR-29 family was attributed to the decrease of Akt2 and CCND2, two key signaling molecules. Inverse correlation among Akt2, CCND2 or c-MYC levels with miR-29b were detected in AML blasts. Furthermore, a feed-back loop comprising of c-MYC, miR-29 family and Akt2 was discovered, further supporting the existence of oncogenic molecular circuitries promoting myeloid leukemogenesis via miR-29b down-regulation [165]. Restoration of miR-29b by transfecting miR-29 oligonucleotides into AML cell lines or primary samples led to a dramatic reduction of tumorigenicity in xenografted AML models [155]. In order to reduce degradation in bio-fluids and increase cellular uptake of miR-29b in AML cells, a novel transferrin-conjugated nanoparticle delivery system for synthetic miR-29b (Tf-NP-miR-29b) was developed by the same group. Of note, this kind of formulation improved miR-29b effects in terms of both down-regulation of oncogenic targets and inhibition of tumor growth, thus supporting the therapeutic value of miR-29b replacement in acute leukemias and the need to develop specific formulations to improve the biodisponibility of microRNAs into body fluids [166]. Studies in AMLs have provided a wide scenario supporting the role of miR-29b as epi-miRNA. Aberrant DNA hypermethylation, mainly due to over-expression of DNMTs, contributes to myeloid leukemogenesis by silencing structurally normal genes involved in hematopoiesis [167]. Evidence of epi-miRNA activity of miR-29b has been widely provided in AML: as observed in MM, enforced expression of miR-29b in AML cells results in marked reduction of the expression of DNMT1, DNMT3A, and DNMT3B at both RNA and protein levels; this in turn led to decrease in global DNA methylation and re-expression of tumor suppressors p15(INK4b) and ESR1 through promoter DNA hypomethylation. Although down-regulation of DNMT3A and DNMT3B was the result of a direct interaction of miR-29b with the 3′ untranslated regions of these genes, no miR-29b interaction sites were detected in the DNMT1 3′ UTRs. Further experiments revealed that miR-29b down-regulates DNMT1 indirectly by targeting SP1, a transactivator of the DNMT1 gene [42]. Altogether, these data indicate a potential therapeutic use of synthetic miR-29b oligonucleotides as effective hypomethylating compounds also in the setting of AMLs.

The relevance of the TF SP1 in regulating miR-29b expression has been also proven in AML bearing *KIT* mutations, where SP1 participates in an epigenetic complex formed by NF-kB/HDAC which represses miR-29b transcription. Interestingly, miR-29b oligonucleotides as well as pharmacological re-activation of miR-29b via HDAC-inhibition, exert anti-AML activity in KIT-driven AMLs, therefore providing a novel experimental platform to treat this specific AML clinical subgroup [43]. Another important epigenetic circuitry has been found in large granular lymphocyte (LGL) leukemia, where IL-15 initiates cancer by inducing an HDAC1/c-MYC/NF-kB complex which repress miR-29b expression, thus resulting in increased DNMT expression and DNA hypermethylation [168].

On the basis of its hypomethylating activity, authors validated miR-29b levels as predictive of response to decitabine in AML patients. To this aim, a phase II clinical trial with single-agent decitabine was carried out in older patients with previously untreated AML not eligible for or who refused intensive chemotherapy. The complete remission rate was 47%, achieved after a median of three cycles of therapy, which raised to 64% if considering nine additional subjects who had no morphologic evidence of disease with incomplete count recovery. Complete remission was achieved in 50% of subjects presenting with either normal or complex karyotypes. Importantly, higher levels of miR-29b were associated with clinical response, supporting the role of miR-29b in predicting response of older AML patients to decitabine [169]. These findings prompted the same authors to exploit other therapeutic strategies up-regulating miR-29b levels in order to increase decitabine response in AML. Interestingly, the HDAC inhibitor AR-42 induced miR-29b levels, this effect being accompanied by down-regulation of known miR-29b targets (DNMT3A, DNMT3B, SP1). Priming of AML cells with AR-42 before decitabine treatment resulted in a stronger anti-leukemic activity *in vitro* and *in vivo* than decitabine followed by AR-42 or either drug alone [170], providing the basis of novel treatment approach based on the combination of epigenetic-targeting compounds in AML. Basing on the same premises, the authors explored another miR-29b-inducing compound, i.e. the proteasome inhibitor bortezomib, to potentiate decitabine response in AML patients. To this purpose, a phase 1 trial of bortezomib and decitabine was carried out. Induction with decitabine plus bortezomib was tolerable, although bortezomib-related neuropathy developed after repetitive cycles. Of previously untreated patients (age ≥ 65 years), 5 of 10 had CR (complete remission, n = 4) or incomplete CR (CRi, n = 1); 7 of 19 overall had CR/CRi. Pharmacodynamic analysis showed FLT3 down-regulation on day 26 of cycle 1. Elegant mechanistic studies showed that FLT3 down-regulation was due to bortezomib-induced miR-29b up-regulation, that led to SP1 down-regulation and destruction of the SP1/NF-κB complex that transactivated FLT3 [171]. This study demonstrated for the first time the feasibility and the preliminary clinical activity of decitabine plus bortezomib combination treatment in AML.

### Chronic myeloid leukemia

The relevant role of miR-29 family in AML raised the question whether these miRNAs may be in involved in the pathogenesis of chronic myeloid leukaemia (CML). Up to date, few reports have been produced that seem to confirm this hypothesis. Starting from the observation that miR-29a/b are down-regulated in AML, the levels of these miRNAs were analysed in 14 newly untreated CML patients [164] and found consistently suppressed. As a comparison, newly diagnosed AML and healthy people were also analysed. Among the miR-29 targets that can be relevant for CML pathogenesis, BCL-2 and MCL-1 were found up-regulated in CML patients as shown in AML [172]. Emerging findings indicate ABL1 as a new validated target of miR-29b [173]. Enforced expression of miR-29b within K562 cells inhibits cell growth and colony formation and promotes apoptosis through caspase 3 and PARP. However, ABL1 suppression is likely not responsible alone for miR-29b-mediated inhibition of cell growth and induction of apoptosis. Indeed, Lee et al. [174] showed that miR-29a/b/c target RNAse-L in K562, leading to inhibition of cell proliferation. RNAse-L-stable knockdown in K562 inhibited tumorigenesis in a xenograft model, supporting the oncogenic role of RNAse-L in tumor development and progression. Overall these data support a tumor suppressor role of miR-29s in CML.

### Lymphoma

Several reports have evaluated miRNA expression profile in Non Hodgkin Lymphomas (NHLs). Due to the extreme heterogeneity of these diseases, miRNA signatures may significantly differ among these studies. Di Lisio et al. [175] profiled 147 NHLs, that included 12 Burkitt Lymphomas (BL), 29 Diffuse Large B cell Lymphomas (DLBCL), 22 mantle cell lymphomas (MCL), 17 Splenic Marginal Zone Lymphomas (SMZL), 18 CLL, 23 Follicular Lymphomas (FL), 11 Nodal marginal Zone Lymphomas (NMZL) and 15 MZL/MALT, and 15 non tumoral samples. 14 deregulated miRNAs were used for RT-PCR validation. In BLs, miR-29a/b/c were found down-regulated, while in DBLCLs were up regulated. TCL-1 and MCL-1 were proven as relevant targets of miR-29 family that are involved in high grade lymphoma pathogenesis [82, 149]. Down-regulation of miR-29a/b has been also described in SMZLs carrying the 7q32 deletion [176]. Translocations involving chromosomes 3 and 7, that fuse BCL-6 to the non coding region FRAH7, have been reported. FRAH7 contains a miRNA gene cluster that includes miR-29b; indeed, the t(3;7)(q27;q32) leads to miR-29b down-regulation. The relevance of miR-29b suppression on lymphoma outcome has been examined by Zhao et al. [177], who profiled 30 MCL patients. Such analysis again highlighted miR-29b down-regulation and associated low levels of miR-29b with worse OS. The prognostic value of miR-29b was equivalent to MIPI score and better than IPI. The authors identified CDK6 as an important target, that supports MCL development. Based on these data, further light on miR-29b-related mechanisms of lymphomagenesis has been shed by Zhang et al [178]. Importantly, the authors provided evidence of an epigenetic circuitry promoting lymphomagenesis and which is based on miR-29b down-modulation: in detail, miR-29b promoter was found to be targeted by C-MYC, that recruits HDAC3 and PRC2, leading to histone deacetylation and trimethylation and consequent miR-29b suppression. Upregulation of miR-29b can be achieved by inhibition of HDAC3 with vorinostat, of PRC2 by its inhibitor 3-deazaneplanocin, and siRNA-mediated C-MYC silencing. Additional partners such as EZH2 and SUZ12 are recruited by c-MYC and concur to miR-29b down-regulation. Furthermore, MYC contributes to EZH2 up-regulation by inhibiting the EZH2 targeting miR-26a, and EZH2 induces MYC through inhibition of the MYC-targeting miR-494 thus generating a positive feedback loop. Combination therapies based upon vorinostat and 3-deazaneplanocin resulted in lymphoma growth suppression *in vitro* and *in vivo* and restored miR-29 expression, thus leading to inhibition of the lymphoma-associated miR-29 targets, CDK6 and IGF1R. Overall, these findings support the role of miR-29b as a TS- miRNA in lymphomas.

**Figure 2:**
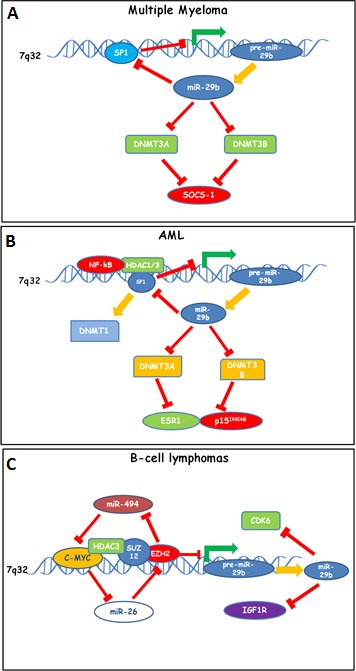
Down-modulation of miR-29b by epigenetic feedback loops sustaining proliferation and survival of (**A**) Multiple Myeloma, (**B**) AML and (**C**) B-cell lymphoma cells. A. In MM cells, SP1 acts by repressing miR-29b promoter thus reducing miR-29b expression; miR-29b in turn targets SP1, DNMT3A and DNMT3B, thus inducing global DNA hypomethylation and reactivation of SOCS-1 by promoter hypomethylation. B. In AML cells, a molecular complex formed by HDAC1-3/NF-kB and SP1 represses miR-29b promoter transcription. In turn, miR-29b targets SP1, a DNMT-1 transactivator, DNMT3A and DNMT3B, thus up-regulating ESR1 and p15 by promoter hypomethylation. C. In aggressive B-cell lymphomas, repression of miR-29b is accomplished by a multimeric complex comprising c-MYC, HDAC3, SUZ12 and EZH2 which represses miR-29b promoter. Of note, EZH2 causes H3K27me3-dependent repression of miR-494, thus up-regulating the miR-494 target c-MYC, which in turn promotes EZH2 expression by inhibiting miR-26. miR-29b-dependent anti-proliferative effects in B-cell lymphomas rely on CDK6 and IGF1R targeting.

Hematologic cancer-related functionally validated targets of miR-29 family members are summarized in Table 2. Epigenetic molecular circuitries involving miR-29b and operative in hematologic cancers are shown as cartoon in Figure 2.

**Table 2:** Functionally validated miR-29 targets in hematologic neoplasias

Target	Neoplasia	miR-29 family member	Reference
MCL-1	MM, AML	miR-29b	[27] [155]
CDK6	MM, AML, B-cell lymphomas, MCL	miR-29a/b/c	[27] [155] [177]
CXXC6	AML	miR-29b	[42]
TCL-1	CLL	miR-29b	[149]
SP1	AML, MM	miR-29b	[42] [28]
DNMT3A/B	AML, MM	miR-29b	[42] [28]
AKT2	AML	miR-29a/b/c	[165]
CCND2	AML	miR-29a/b/c	[165]
ABL1	CML	miR-29b	[173]
RNASE-L	CML	miR-29a/b/c	[174]
PSME4	MM	miR-29b	[131]
MMP2	MM-BD	miR-29b	[30]
c-FOS	MM-BD	miR-29b	[30]
CCNT2	AML	miR-29a	[161]
IGF1R	B-cell lymphomas	miR-29a/c	[178]

Abbreviations: AML, Acute Myeloid Leukemia; CLL: Chronic Lymphocytic Leukemia; CML, Chronic Myeloid Leukemia; MCL: Mantle Cell Lymphoma; MM, Multiple Myeloma; MM-BD, Multiple Myeloma-related Bone Disease.

## PERSPECTIVES AND CONCLUSIONS

The comprehension of the molecular machinery which orchestrates cancer development and progression is critical for diagnosis, prognosis and treatment. Although highly heterogenous in their regulatory mechanisms, alterations of epigenetic processes are emerging as a relevant unifying theme in hematologic malignancies [179], which actually represent excellent models to investigate epigenetic pathways in the challenging quest for more effective anti-cancer therapeutics. A plethora of data have been generated in the last decade regarding ncRNAs, their regulation by the epigenetic machinery and, in turn, their role in the regulation of epigenetic processes [179]. Understanding how these two mechanisms of regulation are integrated in disease pathobiology is disclosing new avenues for the identification of suitable predictive and prognostic tools for personalized therapies. In this scenario, epi-miRNAs are emerging as excellent candidate epi-therapeutics for their prominent role in the regulation of the epigenetic machinery. Several reports indicate that miR-29 family members are TS in almost all hematological malignancies, in which their activity is often associated to inhibition of key epigenetic effectors, such as DNMTs or polycomb genes, and to re-activation of epigenetically-silenced anti-oncogenic pathways [179]. In addition, the relevance of the TS role of miR-29s is further underlined by the onset of cancer-associated epigenetic circuitries which indeed require miR-29 inhibition for driving malignant transformation (Figure 2). Preliminary indications demonstrate that miR-29 might target TET enzymes in solid cancers [180], although data in hematologic diseases such as AML, where TET family members do play a role, are still lacking, thus suggesting that the search of additional epigenetic targets underlying epi-miR-29 activity should go ahead and would possibly contribute to better design miR-29-based epi-therapeutic approaches.

On the light of data presented in this review, synthetic miR-29 mimics represent novel epi-therapeutics since they are able to inhibit MM and AML growth in suitable pre-clinical models, and these effects likely rely on the induction of global DNA hypomethylation and re-activation of promoter-hypermethylated tumor suppressor genes both *in vitro* and *in vivo* [179]. Overall, miR-29b mimics would seem candidates to enter clinical trial in hematological cancers. Undoubtedly, one of the main advantages of miRNA-based therapy is the fact that miRNAs have the ability to simultaneously target several cellular pathways, although this intrinsic multi-target property could potentially result in off-target side effects [179]. Systemic overexpression of miR-29s, which regulate physiological processes within the hematopoietic compartment, could likely target genes in healthy tissues. Moreover, it has to be considered that some studies have reported an oncogenic activity of miR-29 family members in certain hematologic [160] and solid [181, 182] tumors, although results seem controversial and additional preclinical studies are required. Hence, before entering the clinical arena, much work needs to be done in order to clarify the pathophysiological role of miR-29 members in the context of murine models which better recapitulate the scenario of the human disease. MiRNA-based treatment is currently becoming a reality and biotech companies are focusing on the use of miRNAs as novel theranostics, while pharmaceutical companies are finalizing preclinical research towards clinical trials. Importantly, the translation into clinics requires the development of safe delivery vehicles which allow systemic transport of oligonucleotides to tumor tissues [183]. Expanding the knowledge on the mechanisms that facilitate the distribution of miR-29 oligonucleotides in tissues, their stability in biological fluids and their selective uptake by cancer cells to achieve sustained target inhibition, would provide relevant information which will increase knowledge on RNA-based therapeutic approaches and will hopefully lead to the clinical development of miR-29 therapeutics to cure hematologic malignancies.

### BOX-1. DNA-methylation

DNA methylation, i.e. the covalent addition of a methyl-group on the 5′ cytosine residue (5mC) preceeding a guanine, is the reaction catalyzed by DNA methyltransferases (DNMTs) which utilizes S-adenosyl-L-methionine (SAM) as methyl donor. Three different DNMT isoforms, DNMT1, DNMT3A and DNMT3B, have been so far identified and characterized: DNMT3A and DNMT3B, which account for *de novo* DNA methylation, and DNMT1, which is responsible for replicating the DNA methylation pattern in genomic DNA [204].

CpG dinucleotides are enriched in CpG islands located at the 5′ flanking promoter regions of genes, close to their transcriptional start site (TSS) [205]. The outcome of DNA methylation is dependent on the location of methylation sites within a gene. CpG poor-promoters are also subjected to DNA methylation close to their TSS and like in CpG-rich promoters; this negatively correlates with gene expression. However, CpG sites are also found within gene coding sequences and methylation of these sites positively correlates with gene expression [206]. In general, human tumors are globally hypomethylated and this contributes to genomic instability, transposon activation and accumulation of mutations [205]. However, locus-specific hypermethylation occurring at CpG islands located in the promoter region of TS genes, have been detected in many hematologic neoplasias like AML [207] and MM [133], where it is responsible for gene silencing. This event is frequent for genes involved in cell cycle regulation, cell invasion, growth factor signaling, DNA repair and immune modulation. Methylation of several of these genes, like SPARC, BNIP3 [208], TGFβR2 [179] and p16 has been associated with poor prognosis of MM patients [133] or, as for SOCS-1, with the progression from MGUS to MM [209].

Another group of enzymes involved in DNA methylation is represented by *TET* (*Ten-Eleven-Translocation*) family, which comprises three enzymes able to convert 5-methylcytosine (5-mc) to 5-hydroxymethylcytosine (5-hmc) [210]. 5mC can be recognized by proteins containing methyl binding domains (MBD), thus resulting in the recruitment of proteins which trigger repressive histone modifications and chromatin remodeling. DNA demethylation is carried out by enzymatic activity and includes conversion of 5mC by deamination to thymine (catalyzed by AID) or by hydroxylation to hydroxyl-methyl cytosine (5hmC; catalyzed by the TET family). The function of 5-hmc is still under debate, although it has been hypothesized to be an intermediate in the demethylation of DNA and to play a role in base excision repair mechanisms [211]. TET2 mutations, generally resulting in gene inactivation, have been found in AML, MDS, myeloproliferative neoplasms and CML, and generally associate with other myeloid-related gene mutations; TET1 was instead found as MLL fusion partner in AML patients bearing the t(10;11)(q22;q23) translocation [212]. Aberrant DNMT activity might be pharmacologically inhibited: 5-azacitidine and 5-aza-deoxycitidine (decitabine) are two FDA-approved DNA hypomethylating agents which have proven efficacious in many types of cancers. The mechanism of action of these drugs seems related to their capacity to intercalate into DNA or RNA at CpG dinucleotides and to trap DNMTs leading to their degradation and consequent gene hypomethylation and re-expression of silenced loci [213]. The highest therapeutic effectiveness of these two drugs is against MDS and AML.

### BOX-2. histone modifications

Post-translational modifications at the *N-terminal* tails of histones are the result of a complex interplay between different molecules referred to as chromatin modifying proteins which are generally categorized in three different groups: writers, readers and erasers. Writers are enzymes that catalyze the modification while readers are proteins that contain domains recognizing the different types of modifications. Methylated residues can be recognized by PHD fingers and the Tudor-royal family containing tudor domains, chromodomains and MBT domains. Acetylated residues are recognized by bromodomains and phosphorylated residues by a domain in 14-3-3 proteins. Erasers consist of enzymes that remove histone modifications. Histone modifications generally relie upon specific protein complexes consisting of different writers, erasers and chromatin accessory proteins.

#### Histone acetylation

Acetylation of lysine (K) residues of histone tails affects the interaction of histone tails and DNA in nucleosomes. Hyperacetylation results in a more relaxed state of chromatin which enhances accessibility of the transcription machinery; conversely, hypoacetylation leads to a more compact chromatin state and generally leads to gene silencing. The balance of histone acetylation depends on the activity of two enzyme groups: histone acetyltransferases (HATs) and histone deacetylases (HDACs). HDAC proteins can be divided into four classes: class I HDACs (HDAC-1, -2, -3 and -8) are exclusively found in the nucleus, class II (HDAC-4, -5, -6, -7, -9 and -10) shuttle between nucleus and cytoplasm and contain two deacetylase domains. HDAC 11 represents class IV because of the low sequence similarity with other HDACs. Class I, II and IV HDACs all require Zn^2+^ for their catalytic activity. In contrast, class III HDACs (also called sirtuins) are NAD^+^ dependent [214]. Histone tails contain several lysine residues that have been described to be acetylated. Acetylation sites linked with transcriptional activation are for example histone-3-lysine-9 acetylation (H3K9ac), H3K14ac, H3K18ac and H4K5ac. Other functions are also mediated by histone acetylation such as DNA repair (H4K8ac, H3K56ac) and chromatin remodeling (H4K16ac, H2BK12ac) [215]. Along with histones, various other types of proteins involved in transcription, translation, splicing, DNA repair, cell cycle progression, protein folding, cytoskeleton dynamics, signal transduction and metabolism are subjected to acetylation. Protein acetylation regulates several functions including DNA-binding, activity of transcription factors, subcellular localization and protein stability [216].

HDAC inhibitors (HDACi) act by promoting the retention of acetyl groups on histone tails, thus allowing a more active and open chromatin conformation. Most of the HDACi interfere with Zn^2+^ in the catalytic site of one or multiple HDACs. Vorinostat was the first HDACi to enter the clinical arena after its FDA-approval in 2006 for the treatment of cutaneous T-cell lymphoma [217]. Other HDACi, such sodium phenylbutyrate, have given rise to second-generation compounds such as entinostat and panobinostat, which have shown limited efficacy as single agents in MDS, AML, ALL [218] and MM [219].

#### Histone methylation

Histone methylation has been linked to both activation and repression of transcription. Both lysine and arginine (R) residues in histone tails are subjected to methylation. These residues can be mono-, di-, or tri-methylated (only K). The enzymes responsible for histone methylation are grouped in lysine (KHMT) and arginine histone methyltransferases (PRMT) and use SAM as cofactor. The SET domain in KHMTs is responsible for the enzymatic activity. Lysine methylation sites that are linked with transcriptional activation are di-, tri-methylation of H3K4 (H3K4me2/3), H3K36me2/3 and H3K79me, while repressive marks include H3K9me2/3, H3K27me2/3 and H4K20me3 [20,29]. Arginine residues on both H3 and H4 histone can be methylated by PRMTs and include H3R2, H3R17, H3R26 and H4R3. Functionally, arginine methylation can regulate transcriptional activation and repression, mRNA splicing, DNA repair and signal transduction. Histone methylation is reversible as evidenced by the discovery of histone demethylases, such as lysine specific demethylases (KMD1-5, also known as LSD-1) and JumonjiC (JmjC)-domain containing demethylases [220].

Histone modifications may also contribute to determine the global architecture of chromatin. This is demonstrated by the existence of hetero- and euchromatin, each with their specific epigenetic marks. Heterochromatin contains permanently silenced genes and compacted regions such as centromeres and telomeres; typical heterochromatin marks include low levels of acetylation and methylation of H4K9, H3K27 and H4K20. Euchromatin is a less compacted region containing active genes. Genes that are actively transcribed contain high levels of acetylation, H3K4me3 and H3K36me3 [214]. Enhancer of Zeste 2 (EZH2, KMT6) is the catalytic subunit of the Polycomb Repressive Complex 2 (PRC2), which catalyzes dimethylation and trimethylation of K27 on histone H3, which in turn leads to gene repression. EZH2 is found to be over-expressed in a wide variety of cancers where it enhances self-renewal, cell migration and genomic instability [221]. The S-adenosylhomocysteine hydrolase inhibitor 3-deazaneplanocin A (DZNeP) is an EZH2 inhibitor which promotes apoptosis by inducing degradation of EZH2 and consequent reduced levels of H3K27me levels [222]. SUZ12 and EED are other members of the PRC2 complex found mutated in myeloid malignancies [223]. MM carrying the t(4;14) translocation overexpress the MMSET/NSD2/WHSC1 protein, a histone methyltransferase specific for demethylation of lysine 36 on histone H3 (H3K26me2). Overexpression of MMSET leads to a global increase in K36me2 and a decrease in H3K27me2/3 across the genome [224].
